# How much preoperative ROM and FC is a predictor for poor result after total knee arthroplasty in hemophilic arthropathy?

**DOI:** 10.3389/fsurg.2025.1550166

**Published:** 2025-02-11

**Authors:** Hongyu Jiang, Xueke Chang, Fubin Yu, Wei Li, Naihan Fang, Jianzhi Man, Kangshu Guo, Hongzheng Meng, Wenqiang Zhang

**Affiliations:** ^1^ Innovative Institute of Chinese Medicine and Pharmacy, Shandong University of Traditional Chinese Medicine, Jinan, Shandong, China; ^2^ Department of Bone and Joint Surgery, First Affiliated Hospital of Shandong First Medical University, Jinan, Shandong, China; ^3^ Department of Bone and Joint Surgery, Zhangqiu District People’s Hospital, Jinan, Shandong, China; ^4^ Postgraduate Department, Shandong First Medical University, Jinan, Shandong, China

**Keywords:** patients with haemophilia, range of motion, flexion contracture, total knee arthroplasty, knee function

## Abstract

**Aim:**

We aimed to explore the threshold of preoperative range of motion (ROM) and flxion contacture (FC) as a predictor of Poor knee function after TKA.

**Method:**

A retrospective analysis was conducted on 84 Patients with haemophilia (PWH) (113 knees) who underwent knee arthroplasty in our hospital from January 2010 to December 2020 (mean follow-up 70.7 ± 22.4 months). General information, hemophilia information, surgical information, follow-up information were collected. Knees were divided into two groups: Group poor (41 knees) and Group excellent (72 knees). In the clinical evaluation, the FC, ROM and American Society Knee clinical Score (KSC), American Society Knee functional Score (KSF), Hospital for Special Surgery (HSS) scores were used preoperatively and at the final follow-up visit. Receiver operating characteristics (ROC) analysis was used to analyze the threshold of preoperative ROM and FC as a predictor of Poor knee function after TKA.

**Result:**

Postoperative knee joint KSC, KSF, and HSS scores, as well as ROM and reduction in FC deformities at the last follow-up, improved significantly compared to preoperative levels. A notable correlation was observed between ROM and FC and the outcome of knee arthroplasty. The cutoff value of preoperative fexion contracture and ROM for poor knee function at last-follow up was 16.5° and 61.5°.

**Conclusion:**

The study concludes that the efficacy of knee joint replacement surgery in hemophilia patients is influenced by the pre-ROM and Pre-FC. The better the pre- ROM, the better the knee efficacy. The greater the pre- flexion contracture degree, the worse the knee efficacy.

## 1 Introduction

Hemophilia, a blood coagulation disorder caused by a deficiency of coagulation factors, predominantly manifests as bleeding of varying degrees and locations. The most common being sites are the limb joints, particularly the knee joints, which constitute about 45% of all joint bleeding incidents (1). Repeated intra-articular hemorrhage leads to articular cartilage destruction, joint space narrowing, reduced joint mobility, joint deformity, and ultimately, haemophilic arthropathy. The increased bleeding duration, continual iron deposition, and ongoing damage to synovial tissue and cartilage result in chronic proliferative synovial inflammation, exacerbating articular cartilage destruction (2, 3).

Because of postoperative pain and muscle atrophy, rehabilitation training is more arduous for patients with haemophilia (PWH), with recovery being inferior to that of osteoarthritis patients. Prosthesis survival post-surgery is lower in PWH compared to non- hemophiliac individuals, such as older patients with primary osteoarthritis (4, 5). Possible reasons include younger age at the time of surgery, higher activity, heavy wear of the prosthesis, and higher incidence of postoperative infection.

The average range of motion (ROM) of PWH after total knee replacement is less than that in cases of patients with osteoarthritis. Intra-capsular fibrotic changes and extracapsular muscle contractures are typical problems that affect the operative outcomes after TKA (6). The flexion contracture (FC) of the knee joint, especially its rapid progression, is one of the important indication criteria for TKA surgery. The level of postoperative contracture is important for successful outcomes of surgery as well.

Morphologically, these patients often exhibit developmental deformities of the tibial plateau, a reduced aspect ratio of the plateau, flattening of the intercondylar eminence, early formation of large osteophytes on the tibial plateau, altered positioning of the tibial tuberosity, and square patella cartilage formation, accompanied by extensive arthrofibrosis (7). Additionally, joint deformity often leads to a small fronto-occipital diameter and a larger right-left diameter of the femoral and tibial sides, complicating the prosthetic coverage of articular osteotomy surfaces and the intraoperative selection of femoral prosthesis size, as well as determining anatomical markers for the tibial component in tibial plateau replacement (8–10).

Considering the aforementioned studies, it is evident that PWH present greater surgical challenges and generally poorer postoperative outcomes, despite representing a small proportion of all knee replacement surgeries.

The objective of this study is to (1) compare the preoperative and postoperative knee function after total knee arthroplasty (TKA) in HA and, (2) determine the threshold of preoperative range of motion (ROM) and flexion contracture (FC) as a predictor of Poor knee function after TKA.

## 2 Materials and methods

### 2.1 Inclusion and exclusion criteria

Inclusion criteria were: (1) Patients with a confirmed diagnosis of hemophilia who underwent knee replacement; (2) Patients receiving total knee replacement at the First Affiliated Hospital of Shandong First Medical University. Exclusion criteria included: (1) Patients lacking essential information such as demographics, laboratory, and surgical data; (2) Patients with concurrent conditions like tumors; (3) Patients without minimal 4-year follow-up.

### 2.2 Clinical evaluation

All surgeries were conducted by the same department's physician team. PWH were classified into type A and B based onbased on coagulation factor deficiency. Patients were categorized into mild hemophilia (5%–40% coagulation factor activity), moderate (1%–5%), and severe (<1%) groups according to severity. Patients were categorized based on their coagulation factor usage into on-demand and prophylactic treatment groups. Postoperative knee joint outcomes were classified as excellent or poor. The excellent category was defined by scores of 70 or higher on the HSS, KSC, and KSF, a final follow-up ROM of at least 90°, FC under 15°, satisfactory incision healing, absence of infection, exudation, non-healing or delayed healing wounds, and no need for postoperative revision. Patients failing to meet these standards were placed in the poor category.

### 2.3 Perioperative management

According to the Expert Consensus on Management Guide for Hemophilia (11), the FVIII level should be kept at 80%–100% on the operation day, 60%–80% on 1–3 days after the operation, 40%–60% on 4–6 days after the operation and 30%–50% on 7–14 days after the operation. All patients had no autologous blood transfusion. If the patient has HB <70 g/L or HCT <25%, it is considered as an indication that allogeneic blood transfusion is needed (12). Pain control was managed using intravenous narcotics by an intravenous patient controlled analgesia line and non-steroid anti-inflammatory drugs.

### 2.4 Surgical approach

**Figure 1:**
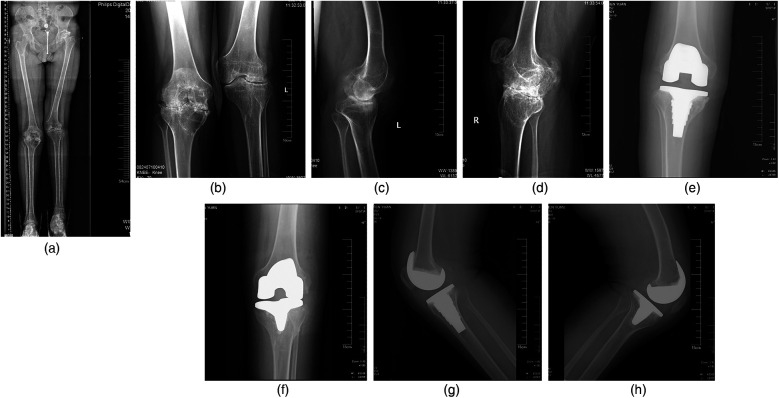
Radiographs of a 28-year-old male patient with severe haemophilic arthropathy. Preoperative x-rays showed extensive destruction of an articular surface, narrowing of the joint space, and a square patella. All operations are performed using mechanical alignment methods. **(a)** Preoperative anteroposterior radiographs of lower extremity length; **(b)** preoperative anteroposterior radiograph; **(c,d**) preoperative lateral radiograph of affected knee joint; **(e,f)** postoperative coronal knee radiographs; **(g,h)** postoperative sagittal knee radiographs.

All PWH underwent a medial parapatellar approach, receiving intravenous tranexamic acid (TXA) (15 mg/kg) 10 min before skin incision and local application of TXA (1 g) in each joint post-joint capsule closure. Antibiotic prophylaxis was administered 30 min before and after surgery, using intravenous cefazolin (2.0 g) at regular intervals, or clindamycin for those allergic to cephalosporin. All operations are performed using mechanical alignment methods (Figure 1). To reduce postoperative bleeding and prevent infection, no drains were used. Pain management included intraoperative joint medication and postoperative oral tramadol (50 mg/bid). General anesthesia was employed, and the prosthesis was fixed with bone cement intraoperatively, akin to conventional total knee replacement procedures.

### 2.5 Postoperative rehabilitation

PWH did not receive anticoagulant therapy post-surgery. Physical methods, including ankle pumps and lower extremity elastic bandages, were employed to prevent deep venous thrombosis in the lower extremities. Patients engaged in exercises such as straight leg raises, ankle flexion, and extension. Continuous passive motion was used routinely, and an intensive rehabilitation protocol with active assisted range of motion exercises was encouraged on the first postoperative day and continued twice daily.

### 2.6 Data analysis

Data were analyzed using SPSS version 30.0. Preoperative and last follow-up values were compared using the *t*-test and chi-squared tests. In all analyses, *p* < 0.05 was considered statistically significant. The relationship between Preoperative values of FC, ROM and knee scores was investigated through the use of correlation analysis. Through receiver operating characteristics (ROC) analysis, we aimed to determine the threshold of preoperative ROM and FC as a predictor of Poor knee function after TKA.

## 3 Results

### 3.1 Patient demographics

A total of 84 (113 knees) PWH were included in this study. A total of 41 knees in the poor group and 72 knees in the excellent group were included according to the evaluation criteria. The follow-up ranged from 47 to 120 months (mean follow-up 70.7 ± 22.4 months). Table 1 showed a significant difference in the severity grading between the groups; a higher proportion of severe hemophilia was noted in the poor group. All eight patients undergoing quadriceps plasty belonged to the poor group (*p* < 0.001), and a higher proportion of patients in the poor group underwent bilateral knee replacements in a single anesthesia session (*p* = 0.037). The intraoperative bleeding was significantly higher in the poor group than in the excellent group. No significant differences were found in the Age, BMI, hemophilia type, Coagulation factor usage, Total Bleeding, Intraoperative blood transfusion or Length of hospital stay.

**Table 1:** Patient demographics.

	Excellent group	Poor group	*P*
Age	33.1 ± 9	35 ± 9	0.302
BMI	23.8 ± 3.2	24 ± 4	0.435
Hemophilia typing (A/B)	60/12	34/7	0.956
Severity grading (mild/moderate/severe)	28/19/25	1/8/32	<0.001
Coagulation factor usage (prophylactic treatment/on-demand)	29/43	13/28	0.365
Intraoperative bleeding	525 ± 172.6	665.8 ± 276	0.004
Total bleeding	826.3 ± 401.3	971.7 ± 450.3	0.079
Intraoperative blood transfusion	664.6 ± 307.4	720.7 ± 311.6	0.355
Quadriceps plasty (Yes/No)	0/72	8/33	<0.001
Simultaneous bilateral knee replacement (Yes/No)	21/51	21/20	0.037
Length of hospital stay	18.9 ± 2.6	19.1 ± 2.4	0.846

ROM, range of motion; FC, flexion contracture.

### 3.2 Clinical results

**Figure 2:**
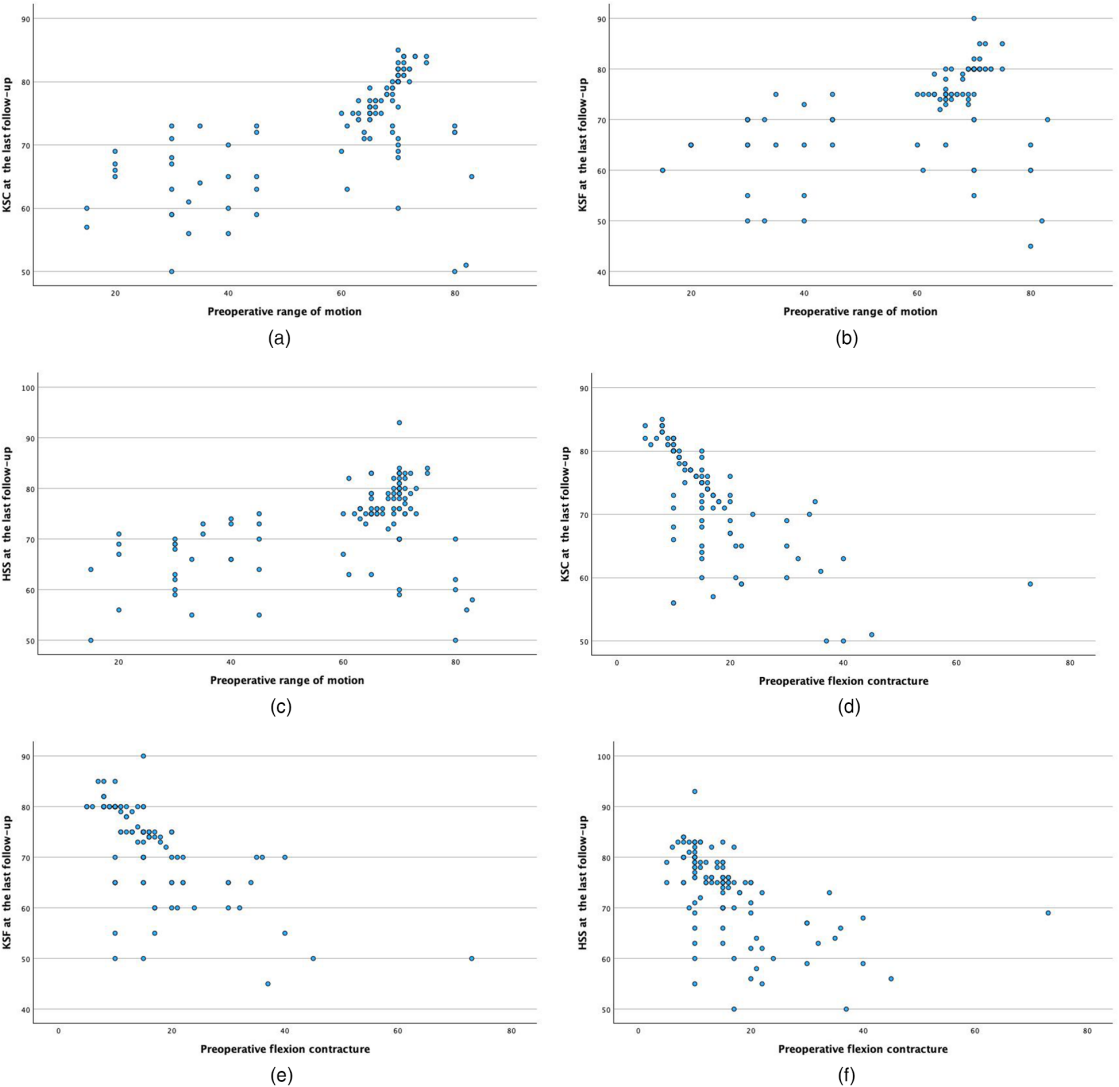
Correlation analysis between preoperative FC, ROM and last follow-up KSC, KSF, HSS. **(a)** Correlation analysis between pre-ROM and last follow-up KSC(*P* < 0.001); **(b)** correlation analysis between pre-ROM and last follow-up KSF(*P* < 0.001); **(c)** correlation analysis between pre-ROM and last follow-up HSS(*P* < 0.001); **(d)** correlation analysis between pre-FC and last follow-up KSC(*P* < 0.001); **(e)** correlation analysis between pre-FC and last follow-up KSF(*P* < 0.001); **(f)** correlation analysis between pre-FC and last follow-up HSS(*P* < 0.001).

**Figure 3:**
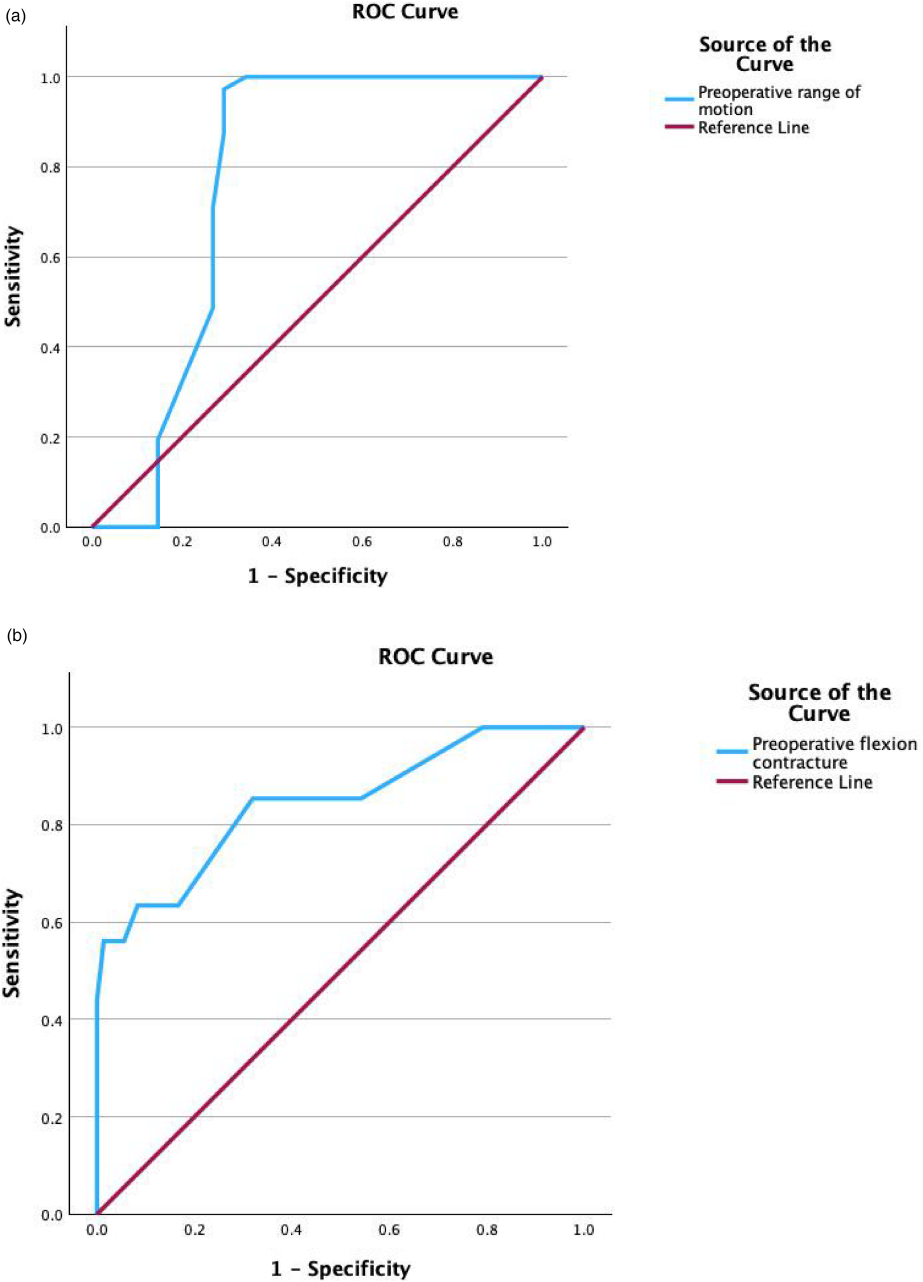
ROC curve to determine the threshold of preoperative ROM and FC as a predictor of poor knee function after TKA. **(a)** The threshold of preoperative ROM as a predictor of Poor knee function after TKA [Area Under the ROC Curve (AUC) = 0.767 Cutoff = 61.5°]. **(b)** The threshold of preoperative FC as a predictor of Poor knee function after TKA [Area Under the ROC Curve (AUC) = 0.843 Cutoff = 16.5°].

The FC decreased from 15.9° ± 9.5° to 7.9° ± 5.8°. The ROM increased from 60.2° ± 16.8° to 74.7° ± 9.7°. Similarly, the KSC, KSF and HSS greatly improved (*P* < 0.001). Thus, there were significant differences between the preoperative and final follow-up values of FC, ROM, KSC, KSF, and HSS, as shown in Table 2. Table 3 showed the degree of ROM at last follow-up was greater in excellent group (79.4° vs. 66.5°, *p* < 0.001). The degree of flexion contracture at last follow-up was greater in the poor group (11.9° vs. 5.6°, *p* < 0.001). The degree of ROM change and FC change was greater in poor group (ROM change 19.4° vs. 11.1°, *p* < 0.001; FC change 11.2° vs. 6.4°). The degree of preoperative ROM and FC was greater in excellent group (Pre-ROM 68.2° vs. 46.2°, *p* < 0.001; Pre-FC 12° vs. 23°). The preoperative and the last follow-up KSC, KSF and HSS scores were greater in the excellent group in comparison to the poor group. KSC, KSF, HSS was positively correlated with preoperative ROM (*P* < 0.001); KSC, KSF, HSS was negatively correlated with preoperative FC(*P* < 0.001) (Figure 2). When evaluating the cutoff point of preoperative FC and ROM for Poor knee function after TKA at last follow-up on ROC analysis, the cutoff value was 61.5° in the ROM and 16.5° in FC (Figure 3).

**Table 2:** Comparison of pre- and post-operative knee function in PWH.

	Preoperative	Postoperative	*P*
KSC	38.6 ± 9.6	73.6 ± 8.2	<0.001
KSF	36.5 ± 10.7	72.9 ± 8.8	<0.001
HSS	37.9 ± 10.7	73.4 ± 8.2	<0.001
ROM	60.2 ± 16.8	74.7 ± 9.7	<0.001
FC	15.9 ± 9.5	7.9 ± 5.8	<0.001

HSS, hospital for special surgery knee score; KSC, American Knee Society's Clinical scores; KSF, American Knee Society's Functional scores.

**Table 3:** Clinical result.

	Excellent group	Poor group	*P*
Pre-KSS	42.2 ± 6.3	32.4 ± 11.1	<0.001
The last follow-up KSS	78.6 ± 3.5	64.8 ± 6.6	<0.001
Pre-KSF	40.8 ± 8.5	29.1 ± 10	<0.001
The last follow-up KSF	78.2 ± 3.4	63.5 ± 7.5	<0.001
Pre-HSS	39.7 ± 9.3	34.7 ± 12.3	0.027
The last follow-up HSS	78.3 ± 3.7	64.8 ± 6.5	<0.001
The last follow-up FC	5.6 ± 2.9	11.9 ± 7.4	<0.001
Pre-FC	12 ± 3.4	23 ± 12.4	<0.001
FC change	6.4 ± 3.6	11.2 ± 7.1	<0.001
Pre-ROM	68.2 ± 3.3	46.2 ± 21.4	<0.001
The last follow-up ROM	79.4 ± 3.4	66.5 ± 11.7	<0.001
ROM change	11.1 ± 5.2	19.4 ± 13	<0.001

ROM, range of motion; FC, flexion contracture.

## 4 Discussion

Knee replacement has been demonstrated to be an effective intervention for improving knee function, knee scores, and knee mobility. Atilla et al. followed 21 patients, utilizing the Knee Society Score (KSS) to evaluate functional and pain levels, noting an increase in ROM from 26.7° preoperatively to 73.0° postoperatively and a decrease in flexion contractures from 21.7° to 8.3°, with the KSS improving from 22.9 to 72.9 points. Pain assessments indicated a significant decrease from 8.4 preoperatively to 2.1 postoperatively, highlighting the procedure's efficacy in enhancing function and alleviating pain in hemophilia patients.

The most important finding of this study is determine the threshold of preoperative ROM and FC as a predictor of Poor knee function after TKA. We strongly advise individuals with hemophilia to prioritize the strengthening of their knee joints by maintaining ROM above 61.5° and FC levels below 16.5°.

Preoperative range of motion (ROM) is usually considered to be the most important predictor of functional outcome after TKA. Atilla et al. also observed that replacements in knees with poor mobility had inferior outcomes compared to those with more flexible joints (13). In a study of 20 patients with haemophilia, the mean ROM at last follow-up was 92° and the mean preoperative flexion contracture was 3° (14). In a cohort of 116 haemophilic procedures at a single institution, a 5° improvement in flexion contracture and a 15° improvement in ROM were reported (15). In our cohort, the average ROM change of the knee joint at the last follow-up was 14.5° and the average FC change was 8°. Compared with the above literature, Our study yielded similar results. This study confirms that knee replacement is effective in managing hemophilic arthropathy.

A study by Noble et al. showed that patients' expectations were highly correlated with their satisfaction after total knee replacement (16). With their serious functional impairment and extreme pain preoperatively, the daily needs of our patients were unfulfilled before TKA. For activities of daily living, 65°–70° of knee flexion are necessary for the swing phase of normal gait, whereas at least 90° are needed to descend stairs and 105° to rise from a low chair (5).

Yi Liu et al. analyzed 71 hemophilia patients (78 knee joints), finding that severely rigid knees (preoperative ROM ≤50°) experienced poorer outcomes and more complications than moderately stiff knees (ROM 50°–90°) (17). These findings are consistent with ours. Other studies have shown a negative correlation between preoperative flexion contracture degrees and postoperative knee function.

Flexion contracture in hemophilic arthropathy is caused from fibrosis of the surrounding soft tissue, combined with periarticular osteopenia (18). Fernandez A et al. reviewed 2,634 knee replacements, finding that contractures over 10° were associated with significantly lower international knee scores and ROM compared to those under 10° (19). Kagaya et al. reported that joint contractures of the lower extremity affect the gait pattern and decrease the patients' ability to walk. They concluded that hip or knee flexion contracture of <15° and absence of ankle equinus is required to maintain positive step length and forward movement of the centre of gravity (20). In the case of flexion contracture following TKA, there are many methods to correct this deformity; such as, manipulation under anesthesia (21), dynamic splinting (22) or revision TKA (23). Our study not only compares the knee function of the excellent group with that of the poor group, but also calculates the exact values of Preoperative ROM and Pre-FC using the ROC analysis.

In summary, ROM and FC are closely related to postoperative knee outcomes, and corresponding functional knee exercises are extremely important. For PWH with a flexion contracture deformity of the knee joint, it is recommended that they apply pressure to the knee joint with a 5-kilogram weight for 10 min, three times a day. In addition, patients should be encouraged to walk more every day, ensuring that they take in more than 5,000 steps. For PWH with severe knee flexion and extension deficits, it is recommended Manipulation under anesthesia.

Concerning knee mobility, Baumgardner et al. (24) identified age as a factor, with mobility decreasing as patients age, possibly due to reduced joint use or immobilization, which leads to quadriceps atrophy and restricts mobility or causes stiffness. Effective postoperative rehabilitation, especially quadriceps training, is crucial as most patients suffer muscle atrophy from inadequate dynamic joint stability, creating a cycle of joint bleeding and exacerbating hemophilic arthropathy. Annual bleeding frequency also impacts mobility, with higher frequencies limiting mobility. Soucie and others have shown that knee function significantly improves when annual bleeding episodes are fewer than 3 (25). Pabinger I advocates for regular administration of concentrated coagulation factors to hemophilia patients (26), while Schramm recommends starting prophylactic treatment for those with 2–3 bleeds per month (27). Prophylactic transfusions and strengthening quadriceps exercises are advised to enhance mobility and improve outcomes following knee replacement surgery.

Obesity is a major health concern not only in the general population but also in patients with haemophilia (28). Body adiposity and disease severity in haemophilia have been found in cross-sectional studies to be negatively associated with joint mobility. Compared with haemophilia males with normal BMI, those who were obese had lower ROM at initial visit and a faster rate of joint mobility loss in the lower limbs (29). We suggest that body weight control and effective treatment of bleeds should be implemented together to achieve better joint ROM outcomes in males with haemophilia.

Research indicates that severity grading is also a significant factor. The age of first bleeding in patients with severe grading hemophilia was significantly younger than that of mild grading patients (*p* < 0.05), and the rate of joint deformity in patients with severe grading hemophilia was statistically different compared to that of mild to moderate grading hemophilia (*p* < 0.05). Severe grading hemophilia patients have a high frequency of bleeding, and the frequency of joint bleeding is an important influencing factor in the formation of hemophilic arthritis. The number of annual bleeds also affects joint mobility, and the degree of restriction of joint mobility is more severe in patients with a high frequency of bleeding (25).

This study had certain limitations. First, this is a retrospective study, with non-flexible knees constituting the control group. Considering the rare incidence of hemophilia, it is difficult to conduct such a prospective study in a single institution. Second, the 5-year follow-up time for some patients was relatively short. Hence, we could not determine the occurrence of late complications, such as aseptic loosening of the prosthesis or late prosthetic joint infection, which potentially develops over a long period after surgery. And the scores used to assess efficacy are subjective. Although the sample size is large for a single institution, including samples from multi-center institutions and expanding the sample size to encompass more influencing factors could provide more definitive results. Despite some limitations, data from the present study can be used to inform PWH about the threshold of poor knee function after TKA.

## 5 Conclusion

The study concludes that TKA can improve knee function in PWH, and the efficacy of knee joint replacement surgery in PWH is influenced by the preoperative ROM and Preoperative FC. The better the preoperative ROM, the better the knee efficacy. The greater the preoperative flexion contracture degree, the worse the knee efficacy. Statistical analysis revealed that preoperative FC of 16.5° and Preoperative ROM of 61.5° is an important threshold. The authors recommend that hemophilia patients actively exercise their quadriceps to enhance knee mobility.

## Data Availability

The raw data supporting the conclusions of this article will be made available by the authors, without undue reservation.
